# The amino acid transporter SLC7A5 confers a poor prognosis in the highly proliferative breast cancer subtypes and is a key therapeutic target in luminal B tumours

**DOI:** 10.1186/s13058-018-0946-6

**Published:** 2018-03-22

**Authors:** Rokaya El Ansari, Madeleine L. Craze, Islam Miligy, Maria Diez-Rodriguez, Christopher C. Nolan, Ian O. Ellis, Emad A. Rakha, Andrew R. Green

**Affiliations:** 1 Academic Pathology, Division of Cancer and Stem Cells, School of Medicine, University of Nottingham, Nottingham City Hospital, Hucknall Road, Nottingham, NG5 1PB UK; 2 Breast Institute, Nottingham University Hospitals NHS Trust, Hucknall Road, Nottingham, NG5 1PB UK

**Keywords:** SLC7A5, Breast cancer, Prognosis

## Abstract

**Background:**

Breast cancer (BC) is a heterogeneous disease characterised by variant biology and patient outcome. The amino acid transporter, SLC7A5, plays a role in BC although its impact on patient outcome in different BC subtypes remains to be validated. This study aimed to determine whether the clinicopathological and prognostic value of SLC7A5 is different within the molecular classes of BC.

**Methods:**

SLC7A5 was assessed at the genomic level, using Molecular Taxonomy of Breast Cancer International Consortium (METABRIC) data (*n* = 1980), and proteomic level, using immunohistochemical analysis and tissue microarray (TMA) (*n* = 2664; 1110 training and 1554 validation sets) in well-characterised primary BC cohorts. SLC7A5 expression correlated with clinicopathological and biological parameters, molecular subtypes and patient outcome.

**Results:**

SLC7A5 mRNA and protein expression were strongly correlated with larger tumour size and higher grade. High expression was observed in triple negative (TN), human epidermal growth factor receptor 2 (HER2)+, and luminal B subtypes. SLC7A5 mRNA and protein expression was significantly associated with the expression of the key regulator of tumour cell metabolism, c-MYC, specifically in luminal B tumours only (*p* = 0.001). High expression of SLC7A5 mRNA and protein was associated with poor patient outcome (*p* < 0.001) but only in the highly proliferative oestrogen receptor (ER)+/ luminal B (*p* = 0.007) and HER2+ classes of BC (*p* = 0.03). In multivariate analysis, SLC7A5 protein was an independent risk factor for shorter breast-cancer-specific survival only in ER+ high-proliferation tumours (*p* = 0.02).

**Conclusions:**

SLC7A5 appears to play a role in the aggressive highly proliferative ER+ subtype driven by *MYC* and could act as a potential therapeutic target. Functional assessment is necessary to reveal the specific role played by this transporter in the ER+ highly proliferative subclass and HER2+ subclass of BC.

**Electronic supplementary material:**

The online version of this article (10.1186/s13058-018-0946-6) contains supplementary material, which is available to authorized users.

## Background

Altered metabolic pathways have been readily accepted as part of the revised hallmarks of cancer where cancer cells are able to regulate their metabolism to provide energy and cellular building blocks required for their unremitting proliferation [1]. Many cancer cells are highly reliant on amino acids for their growth, not only because they are a nitrogen donor for the synthesis of nucleotides and other amino acids, but also because they activate mammalian target of rapamycin complex1 (mTORC1) through nutrient signalling pathways which in turn regulates protein translation and cell growth [2, 3]. There is also increasing evidence that oncogenes and/or tumour-suppressor genes can reprogramme tumour cell metabolism including the direct regulation of the amino acid transporter, solute carrier family 7 member 5 (SLC7A5), by the oncogene *MYC* [4, 5] and the regulation of expression of the glutamine transporter, SLC1A5, by the tumour suppressor retinoblastoma (*Rb*) [6].

SLC7A5 is a sodium-independent transporter and acts as an amino acid exchanger by transporting large neutral amino acids such as leucine, phenylalanine and tryptophan by exchange with intracellular glutamine [7]. It therefore functions in supplying amino acids to cancer cells and maintaining intra-cellular leucine, which is considered a master regulator of the mTORC1 signalling pathway [8–10]. For functional expression on the plasma membrane, SLC7A5 must heterodimerise with the heavy chain of SLC3A2 [7, 11].

It has been reported that SLC7A5 is highly expressed in a variety of cancers including oesophageal carcinoma [12], oral cancer [13] and lung adenocarcinoma [14]. SLC7A5 is co-expressed with the glutamine transporter, SLC1A5, in many cancers suggesting a functional coupling of these transporters in supporting tumour progression [15]. In this study, we aimed to assess SLC7A5 gene copy number and mRNA expression, alongside protein expression in large and well-characterised annotated cohorts of BC to determine its biological, clinicopathological and prognostic value in the different BC molecular classes with particular interest in the highly proliferative aggressive subgroups.

## Methods

### *SLC7A5* copy number and gene expression

A cohort of 1980 BC tumours in the Molecular Taxonomy of Breast Cancer International Consortium (METABRIC) [16] was used to evaluate *SLC7A5* gene copy number aberrations (CNA) and gene expression. DNA/RNA was isolated from fresh frozen samples and genomic and transcriptional profiling was obtained using the Affymetrix SNP 6.0 and Illumina HT-12v3 platforms respectively. CNA were considered at the gene level by segments and the Šidák correction [17] was applied for multiple testing. Gene expression data were pre-processed and normalised as described previously [16]. In this cohort, patients included were oestrogen receptor (ER)-positive (ER+) and/or lymph node (LN)-negative (LN-) and did not receive adjuvant chemotherapy, whereas ER- and LN+ patients received adjuvant treatment. X-tile (version 3.6.1, Yale University, USA) was applied to dichotomise SLC7A5 mRNA expression, based on prediction of breast-cancer-specific survival (BCSS). The association between the SLC7A5 mRNA expression and clinicopathological parameters, molecular subtypes, and patient outcome was investigated. The online dataset, Breast Cancer Gene Expression Miner v4.0 (http://bcgenex.centregauducheau.fr) and breast cancer data from The Cancer Genome Atlas (TCGA) [18] were used for external validation of *SLC7A5* copy number/or mRNA expression.

### Patients and tumours

This study evaluated well-characterised cohorts of patients with early-stage primary operable invasive BC, who presented aged ≤70 years. Patients in the discovery set (*n* = 1110) presented at Nottingham City Hospital between 1989 and 1998, while the validation set (*n* = 1554) includes patients who were presented between 1998 and 2006. Patient management was uniform and based on tumour characteristics by Nottingham Prognostic Index (NPI) and hormone receptor status. Patients within the NPI excellent prognostic group (score ≤3.4) received no adjuvant therapy, but those patients with NPI >3.4 received tamoxifen if ER-positive (± goserelin (Zoladex) in case the patients were premenopausal). Conversely, classical cyclophosphamide, methotrexate and 5-flurouracil (CMF) were used if the patients were ER-negative and fit enough to receive chemotherapy. None of the patients in this study received neoadjuvant therapy. Clinical history, tumour characteristics and information on therapy and outcomes are prospectively maintained. Outcome data included development and time to distant metastasis (DM) and breast-cancer-specific survival (BCSS). There was no difference in the distribution of clinicopathological parameters between the discovery and validation cohorts or the METABRIC series of patients (all correlation coefficients ≥0.91, all *p* < 0.0001) (Additional file 1: Table S1).

### Western blotting

The antibody specificity of anti-SLC7A5 (EPR17573, Abcam, UK) was validated using western blotting in human embryonic kidney (HEK) 293 T over expression lysate (Origene Technologies, Rockville, MD, USA) and MDA-MB-175 (luminal B-like), T47D and MCF7 (luminal A) [19] breast cancer lysate (American Type Culture Collection; Rockville, MD, USA). A dilution of 1:200 of the primary antibody and 1:1000 HRP-conjugated (Dako) secondary antibodies were applied: 5% milk /PBS-Tween (0.1%) (Marvel Original Dried Skimmed Milk, Premier Food Groups Ltd., UK) was used for blocking. Mouse monoclonal anti-β-actin primary antibody was used as a marker of endogenously expressed control. SLC7A5 bands were visualised using enhanced chemiluminescence (ECL) showing a single specific band at the correct predicted size (40 kDa) for the SLC7A5 protein.

### Tissue arrays and immunohistochemical analysis

The discovery set (*n* = 1110) were arrayed as previously described using a single 0.6-mm core sampled from the periphery of each invasive tumour [20]. The validation set (*n* = 1554) were similarly arrayed using a tissue microarray (TMA) GrandMaster (3D Histech). Immunohistochemical (IHC) staining was performed on 4-μm TMA sections using the Novolink polymer detection system (Leica Biosystems, RE7150-K). Briefly, tissue slides were deparaffinised with xylene and rehydrated through three changes of alcohol. Heat-induced antigen epitope retrieval was performed in citrate buffer (pH 6.0) for 20 min using a microwave oven (Whirpool JT359 Jet Chef 1000 W). Endogenous peroxidase activity was blocked by peroxidase block for 5 min. Slides were washed with Tris-buffered saline (TBS, pH 7.6), followed by application of protein block for 5 min. Following another TBS wash, sections were incubated with the primary SLC7A5 antibody diluted at 1:50 in Leica antibody diluent (RE AR9352, Lieca, Biosysytems, UK) overnight at 4 °C. Slides were washed with TBS followed by incubation with post primary block for 30 min followed by a TBS wash. Novolink polymer was applied for 30 min: 3,3′-diaminobenzidine (DAB) chromogen was applied for 5 min. Slides were counterstained with Novolink haematoxylin for 6 min, dehydrated and coverslipped.

Stained TMA sections were scored using high resolution digital images (NanoZoomer; Hamamatsu Photonics, Welwyn Garden City, UK), at × 20 magnification. Evaluation of staining for SLC7A5 was based on a semi-quantitative assessment of digital images of the cores using a modified histochemical score (H-score) which includes an assessment of both the intensity and the percentage of stained cells [21]. Staining intensity was assessed as follows: 0, negative; 1, weak; 2, medium; 3, strong, and the percentage of the positively stained tumour cells was estimated subjectively. The final H-score was calculated by multiplying the percentage of positive cells (0–100) by the intensity (0–3), producing a total range of 0–300. Dichotomisation of protein expression in predicting BCSS was determined using x-tile software.

Immunhistochemical staining and dichotomisation of the other biomarkers included in this study were as per previous publications [20, 22–30]. ER and progesterone receptor (PgR) positivity was defined as ≥ 1% staining. Immunoreactivity of HER2 in TMA cores was scored using standard HercepTest guidelines (Dako). Chromogenic in situ hybridisation (CISH) was used to quantify HER2 gene amplification in borderline cases using the HER2 FISH pharmDx™ plus HER2 CISH pharmDx™ kit (Dako) and was assessed according to the American Society of Clinical Oncology guidelines. BC molecular subtypes were defined based on tumour IHC profile and the Elston-Ellis [31] mitotic score as: ER+/HER2- low proliferation (mitotic score 1), ER+/HER2- high proliferation (mitotic score 2 and 3); HER2-positive class: HER2+ regardless of ER status; triple negative: ER-, PgR- and HER2- [32]. Basal-like phenotype was defined as tumours expressing cytokeratin (Ck) 5/6, and/or Ck14 and/or Ck17.

### Statistical analysis

Statistical analysis was performed using SPSS 22.0 statistical software (SPSS Inc., Chicago, IL, USA). Spearman’s correlation coefficient was calcualted to examine the association between continuous variables. The chi-square test was performed to analyse relationships between categorical variables. For the continuous variables, differences between three or more groups were assessed using one-way analysis of variance (ANOVA) with the post-hoc Tukey multiple comparison test (for normally distributed data) or Kruskal-Wallis test (for non-normal distribution). Differences between two groups were assessed using the *t* test (normally distributed data) or Mann-Whitney test (non-normal distribution). Survival curves were analysed by the Kaplan-Meier and log rank test. Cox’s proportional hazard method was performed for multivariate analysis to identify the independent prognostic/predictive factors. The *p* values were adjusted for multiple testing using the Bonferroni correction. A *p* value ˂0.05 was considered significant. The study endpoints were 10-year BCSS or distant metastasis-free survival (DMFS).

## Results

### *SLC7A5* genomic profiling

*SLC7A5* was amplified in 0.3% and 0.6% of BC cases in the METABRIC and TCGA datasets, respectively, while deletion (deep and shallow) was detected in 56% and 68% of cases in the same cohorts respectively. Point mutations in *SLC7A5* were extremely rare, where TCGA data reported just one case with a missense mutation (Additional file 2: Figure S4A) [33, 34]. *SLC7A5* is situated on chromosome 16 (16q24.2); all the annotated genes, which were located on 16q [35] were selected to determine their CNV in relation to *SLC7A5* and assess whether these aberrations were locus-specific or involved large chromosomal segments. There was significant positive correlation between *SLC7A5* deletion and the deletion of all genes (*p* < 0.001, Additional file 3: Table S2) in both the METABRIC and TCGA data. However, amplification of three genes (*FANCA*, *CBFA2T3* and *CDT1*) showed significant association with the amplified *SLC7A5* (*p* ≤ 0.03, data not shown) in the afore-mentioned datasets together.

### SLC7A5 expression in breast cancer

**Fig. 1:**
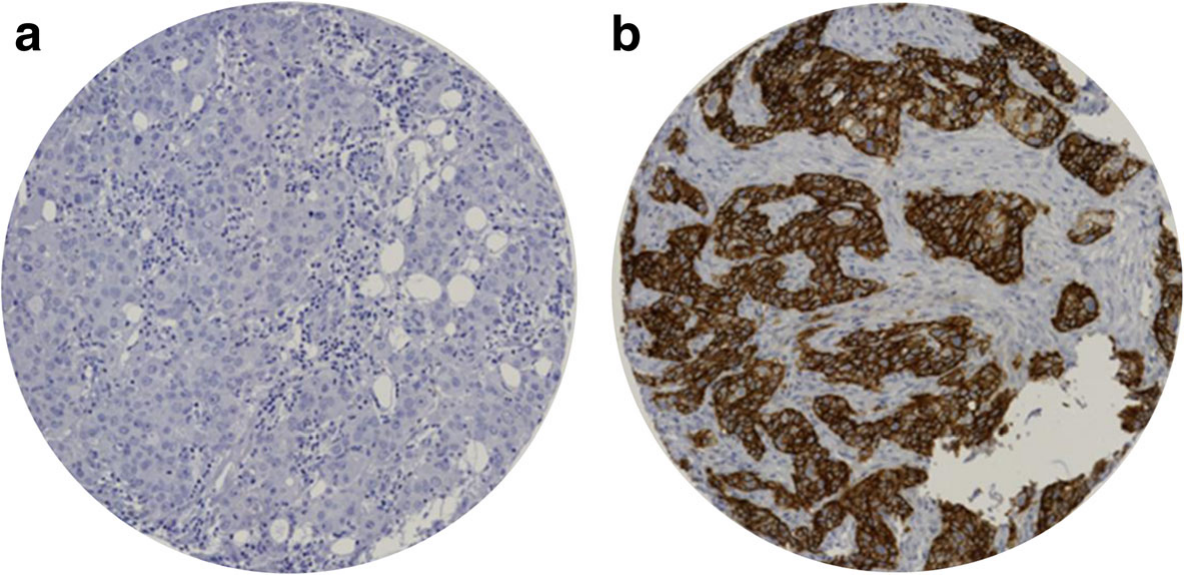
SLC7A5 protein expression in invasive breast cancer cores. **a** Negative immunohistochemical (IHC) expression. **b** Positive IHC expression

**Fig. 2:**
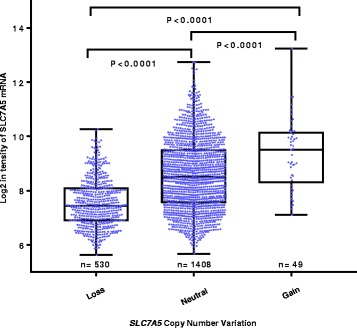
*SLC7A5* copy number aberrations and relationship with mRNA expression in the Molecular Taxonomy of Breast Cancer International Consortium (METABRIC) cohort using one-way analysis of variance and the post-hoc Tukey test

SLC7A5 protein expression was observed, predominantly in the membrane of invasive breast cancer cells, with expression levels varying from absent to high (Fig. 1b and c). The distribution of the SLC7A5 protein expression was unimodal and left-skewed. The SLC7A5 mRNA expression had a normal distribution. Expression of SLC7A5 mRNA and protein were dichotomised using cut points derived from prediction of patient survival using X-tile (https://medicine.yale.edu/lab/rimm/research/software.aspx; Yale University). Positive SLC7A5 expression (>15 H-score) was observed in 191/1110 (17%) and 268/1554 (17%) of cases in the discovery and validation sets, respectively, while high SLC7A5 mRNA expression (log2 intensity >8) was observed in 1019/1923 (53%) of the METABRIC breast cancer cases. A total of 49/1980 (2.4%) of cases had a copy number (CN) gain of *SLC7A5* and 530/1980 (26.7%) a CN loss. Significant association was observed between *SLC7A5* copy number variation (CNV) and SLC7A5 mRNA expression (*p* < 0.001, Fig. 2).

### SLC7A5 and clinicopathological parameters

Table 1 summarises the associations between SLC7A5 protein expression including larger tumour size, high tumour grade, and poor Nottingham Prognostic Index (NPI) (all *p* < 0.001). Regarding BC metastatic sites, high SLC7A5 protein levels were associated with the development of distant metastases (DM) in the brain (*p* < 0.001) and lung (*p* = 0.04), while there was no association with development of DM in the bone or liver.

**Table 1:** Clinicopathological associations of SLC7A5 protein expression in the discovery and validation breast cancer series

SLC7A5 protein										
Discovery set						Validation set				
	Number (%)	Mean		*p* value	Adjusted *p* value	Number (%)	Mean		*p* value	Adjusted *p* value
Patient’s age (years)										
≥50	395 (36)	582.20		0.003	**0.018**	469 (30)	859.46		7.0 × 10^−12^	**<0.0001**
<50	714 (64)	539.95				1070 (70)	730.79			
Tumour size										
≥2.0 cm	532 (48)	521.22		0.000002	**<0.0001**	939 (61)	729.47		6.5 × 10^−9^	**<0.0001**
˂2.0 cm	577 (52)	586.14				599 (39)	832.24			
Lympho-vascular invasion										
Negative	722 (65)	545.17		0.138	0.55	1086 (71)	743.95		0.000007	**<0.0001**
Positive	382 (35)	566.35				451 (29)	829.31			
Site of distant metastasis										
Brain										
No	1044 (94)	546.62		0.0001	**0.0008**	N/A				
Yes	61 (6)	662.27								
Lung										
No	1003 (91)	547.12		0.007	**0.04**	N/A				
Yes	102 (9)	610.80								
Bone										
No	876 (79)	554.57		0.651	1.30	N/A				
Yes	229 (21)	547.00								
Liver										
No	949 (86)	551.43		0.568	1.70	N/A				
Yes	156 (14)	562.57								
	Number (%)	Mean	χ2	*p* value	Adjusted *p* value	Number (%)	Mean	χ2	*p* value	Adjusted *p* value
Tumour grade										
1	190 (17)	450.77	171.5	5.6 × 10^−38^	**<0.0001**	231 (15)	585.30	723.48	7.7 × 10^−72^	**<0.0001**
2	366 (33)	473.19				622 (40)	647.46			
3	550 (50)	642.43				685 (45)	942.43			
Lymph node stage										
1	674 (61)	542.19	4.811	0.09	0.45	955 (62)	754.30	12.56	0.002	**0.004**
2	341 (31)	574.98				428 (28)	767.94			
3	91 (8)	556.75				153 (10)	858.69			
Nottingham Prognostic Index										
Good	332 (30)	458.21	102.4	5.6 × 10^−23^	**<0.0001**	521 (34)	620.66	156.60	9.8 × 10^−35^	**<0.0001**
Moderate	593 (53)	578.27				768 (50)	828.08			
Poor	184 (17)	654.64				246 (16)	892.49			
IHC subtypes										
ER+/HER2- low proliferation	250 (27)	391.92	178.4	1.8 × 10^−38^	**<0.0001**	N/A				
ER+/HER2- high proliferation	351 (38)	419.54								
Triple negative	191 (20)	617.95								
HER2+	143 (15)	519.69								
Histological type										
Ductal (including mixed)	922 (83)	563.83	69.05	3.5 × 10^−14^	**<0.0001**	1335 (87)	782.19	77.07	7.2 × 10^−16^	**<0.0001**
Lobular	101 (9)	454.17				120 (8)	584.17			
Medullary	26 (2)	832.02				13 (0.8)	1257.27			
Miscellaneous	7 (0.6)	440.50				9 (0.6)	1037.78			
Special type	53 (5.4)	472.75				57 (3.6)	655.12			

*IHC* immunohistochemical analysis, *ER* oestrogen receptor, *HER2* human epidermal growth factor receptor 2, *NA* Not applicable

*p* value in bold in these tables means statistically significant associations

**Fig. 3:**
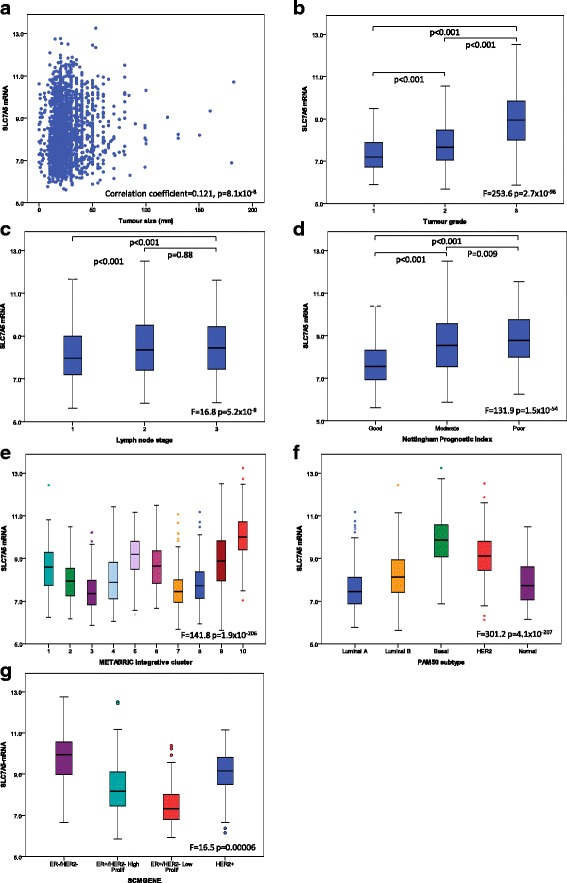
SLC7A5 mRNA expression and its association with clinicopathological parameters and molecular subtypes. **a** SLC7A5 and tumour size. **b** SLC7A5 and tumour grade. **c** SLC7A5 and lymph node stage. **d** SLC7A5 and Nottingham Prognostic Index. **e** SLC7A5 and Molecular Taxonomy of Breast Cancer International Consortium (METABRIC) integrative clusters. **f** SLC7A5 and prediction analysis of microarray 50 (PAM50) subtypes, **g** SLC7A5 and SMCGENE subtypes in the METABRIC cohort using one-way analysis of variance with the post-hoc Tukey test

High SLC7A5 mRNA expression was significantly associated with larger tumour size (Fig. 3a, *p* < 0.001), higher grade (Fig. 3b, *p* < 0.001), positive nodal metastasis (Fig. 3c, p< 0.001) and poor NPI (Fig. 3d, *p* < 0.001). Both SLC7A5 mRNA and SLC7A5 protein were associated with medullary-like tumours. Where data were available, these associations were confirmed using the Breast Cancer Gene-Expression Miner v4.0 (Additional file 4: Figure S1A, B) and the TCGA data (Additional file 2: Figure S4B). In addition *SLC7A5* copy number loss was significantly associated with good prognostic parameters including, lower grade and good NPI (Table 2, *p* < 0.001). There was positive association between *SLC7A5* copy number gain and *MYC* gain (*p* < 0.001, Table 2).

**Table 2:** Copy number aberrations of *SLC7A5* in the Molecular Taxonomy of Breast Cancer International Consortium (METABRIC) breast cancer series and their association with clinicopathological parameters, *MYC* copy number aberrations and breast cancer subtypes

*SLC7A5 copy number*								
Gain				Loss				
	No, number (%)	Yes, number (%)	χ^2^ (*p* value)	Adjusted *p* value	No, number (%)	Yes, number (%)	χ^2^ (*p* value)	Adjusted *p* value
Age (years)								
≥50	1520 (97.7)	36 (2.3)	0.405 (0.524)	1.572	1098 (70.6)	458 (29.4)	27.479 (1.5 × 10^− 7^)	**<0.0001**
˂50	372 (97.1)	11 (2.9)			321 (83.8)	62 (16.2)		
Tumour size								
≥2.0 cm	1291 (97.0)	40 (3.0)	5.226 (0.022)	0.132	976 (73.3)	355 (26.7)	0.094 (0.759)	0.282
˂2.0 cm	614 (98.7)	8 (1.3)			452 (72.7)	170 (27.3)		
Tumour grade								
1	170 (100.0)	0 (0.0)	10.154 (0.006)	**0.042**	99 (58.2)	71 (41.8)	107.36 4.8 × 10^−24^	**<0.0001**
2	756 (98.2)	14 (1.8)			495 (64.3)	275 (35.7)		
3	918 (96.4)	34 (3.6)			799 (83.9)	153 (16.1)		
Lymph node stage								
1	1012 (97.8)	23 (2.2)	0.474 (0.789)	1.578	726 (70.1)	309 (29.9)	10.425 (0.005)	**0.02**
2	606 (97.4)	16 (2.6)			480 (77.2)	142 (22.8)		
3	307 (97.2)	9 (2.8)			237 (75.0)	79 (25.0)		
Nottingham Prognostic Index								
Good	668 (98.2)	12 (1.8)	2.602 (0.272)	0.080	418 (61.5)	262 (38.5)	76.132 (2.9 × 10^−17^)	**<0.0001**
Moderate	1071 (97.3)	30 (2.7)			864 (78.5)	237 (21.5)		
Poor	192 (96.5)	7 (3.5)			168 (84.4)	31 (15.6)		
Histological type								
Ductal	1500 (97.2)	44 (2.8)	6.880 (0.230)	1.150	1154 (74.7)	390 (25.3)	29.544 (0.00001)	**0.0001**
Lobular	145 (98.6)	2 (1.4)			88 (59.9)	59 (40.1)		
Medullary	30 (93.8)	2 (6.3)			30 (93.8)	2 (6.2)		
Miscellaneous	12 (100.0)	0 (0.0)			9 (75.0)	3 (25.0)		
Special type	113 (100.0)	0 (0.0)			74 (66.8)	39 (33.2)		
PAM50 subtype								
Luminal A	710 (98.9)	8 (1.1)	40.515 (3.3 × 10^−8^)	**<0.0001**	423 (58.9)	295 (41.1)	248.3 (1.4 × 10^−52^)	**<0.0001**
Luminal B	477 (97.7)	11 (2.3)			312 (63.9)	176 (36.1)		
Basal	305 (92.7)	24 (7.3)			319 (97.0)	10 (3.0)		
HER2+	235 (97.9)	5 (2.1)			219 (91.3)	21 (8.7)		
Normal-like	198 (99.5)	1 (0.5)			172 (11.9)	27 (5.1)		
*MYC* gain								
No	1228 (98.9)	14 (1.1)	25.0 (5.5 × 10^−7^)	**<0.0001**	1446 (73.3)	528 (26.7)	0.132 (0.716)	1.432
Yes	703 (95.3)	35 (4.7)			4 (66.7)	2 (33.3)		

*PAM50* prediction analysis of microarray, *HER2* human epidermal growth factor receptor 2

*p* value in bold in these tables means statistically significant associations

### SLC7A5 expression in molecular BC subtypes

SLC7A5 protein expression was associated with negative hormone receptor status and HER2+ tumours (all *p* ≤ 0.002, Table 3) and it was highly expressed in triple negative (TN) and basal-like phenotype malignancies compared to non-TN and non-basal-like tumours (*p* < 0.001, Table 3). Similarly, high expression of SLC7A5 mRNA was significantly associated with hormone receptor negative (ER- and PgR-) and HER2+ tumours (all *p* < 0.001, Table 4). These results were in concordance with the findings of the Breast Cancer Gene-Expression Miner v4.0 (Additional file 4: Figure S1C-F) and TCGA data analysis (Additional file 2: Figure S4C-E).

**Table 3:** Association of SLC7A5 protein expression and the expression of other molecular biomarkers in the discovery and validation sets

SLC7A5 protein								
Discovery set					Validation set			
	Number (%)	Mean	*p* value	Adjusted *p* value	Number (%)	Mean	*p* value	Adjusted *p* value
ER								
Negative	270 (25)	722.20	3.2 × 10^−48^	**<0.0001**	300 (19)	1094.70	4.6 × 10^−76^	**<0.0001**
Positive	827 (75)	492.45			1240 (81)	692.06		
PgR								
Negative	435 (41)	619.12	8.8 × 10^−27^	**<0.0001**	612 (42)	855.53	1.3 × 10^−34^	**<0.0001**
Positive	630 (59)	473.54			853 (58)	645.09		
HER2								
Negative	921 (87)	521.78	0.00004	**0.0001**	1337 (92)	718.53	0.001	**0.002**
Positive	143 (13)	601.54			116 (8)	824.67		
Triple negative								
No	896 (83)	503.50	4.5 × 10^−35^	**<0.0001**	1286 (83)	696.76	1.5 × 10^−62^	**<0.0001**
Yes	185 (17)	722.61			225 (17)	1094.6		
Basal phenotype								
No	794 (74)	510.96	6.8 × 10^−13^	**<0.0001**	N/A			
Yes	285 (26)	620.90						
P53 protein								
Negative	760 (72)	499.14	4.1 × 10^−13^	**<0.0001**	N/A			
Positive	298 (28)	606.92						

*ER* oestrogen receptor, *PgR* progesterone receptor, *HER2* human epidermal growth factor receptor, *NA* not applicable

*p* value in bold in these tables means statistically significant associations

**Table 4:** Association of SLC7A5 mRNA expression and the expression of other molecular biomarkers in the Molecular Taxonomy of Breast Cancer International Consortium (METABRIC) series

SLC7A5 mRNA expression					
	Number (%)	Mean	*t* test	*p* value	Adjusted *p* value
Estrogen receptor					
Negative	474 (24)	9.543	26.90	5.6 × 10^− 113^	**<0.0001**
Positive	1506 (76)	7.943			
Progesterone receptor					
Negative	940 (47)	8.862	18.73	1.07 × 10^−71^	**<0.0001**
Positive	1040 (53)	7.841			
HER2					
Negative	1733 (88)	8.216	−12.35	1.1 × 10^−29^	**<0.0001**
Positive	247 (12)	9.095			
Triple negative (ER-, PR-, HER2-)					
No	1660 (84)	8.065	−22.12	1.9 × 10^−73^	**<0.0001**
Yes	320 (16)	9.676			
Basal phenotype					
No	1645 (83)	8.036	−25.70	1.5 × 10^−91^	**<0.0001**
Yes	329 (17)	9.788			
*TP53* mutation					
Wild-type	721 (88)	8.132	−7.47	1.2 × 10^−11^	**<0.0001**
Mutation	99 (12)	9.148			

*ER* oestrogen receptor, *PgR* progesterone receptor, *HER2* human epidermal growth factor receptor 2

*p* value in bold in these tables means statistically significant associations

When comparing the levels of SLC7A5 mRNA expression in the intrinsic (prediction analysis of microarray 50 (PAM50)) subtypes [36], high expression was observed in basal-like, HER2+ and lLuminal B tumours (Fig. 3f, *p* < 0.001). Similarly, within the METABRIC integrative clusters, high SLC7A5 mRNA expression was associated with clusters 5 (ERBB2 amplified), 9 (luminal B subgroup) and 10 (TN/basal-like) (*p* < 0.001, Fig. 3e). In the SCMGENE subtypes there was higher expression of SLC7A5 mRNA in the ER+/HER2- high proliferation class (luminal B) compared with the ER+/HER2- low proliferation class (luminal A) (*p* < 0.001, Fig. 3g). Association between SLC7A5 mRNA and PAM50 subtypes was confirmed using the Breast Cancer Gene-Expression Miner v4.0 (Additional file 4: Figure S1G). There was lower expression of SLC7A5 protein in the low-proliferation tumours than in the other defined molecular subtypes subtypes (*p* < 0.001, Table 1).

At the gene level, there was a greater copy number gain of *SLC7A5* (*p* < 0.001, Table 2 in the basal-like subtype, while *SCL7A5* copy number loss was mainly observed in the luminal A subtype (*p* < 0.001, Table 2).

### SLC7A5 expression and other associated markers

Correlation between SLC7A5 mRNA and associated genes was investigated in the METABRIC dataset (Table 5). The genes were selected based on previous publications, and were either regulatory genes or others that share or support the SLC7A5 biological function, which focused mainly on glutamine transport and glutamine metabolism [2, 5, 15, 37–41]. There was positive correlation between SLC7A5 mRNA expression and the expression of regulatory genes, several amino acid transporters and genes involved in the glutamine-proline regulatory axis. There was a positive relationship between SLC7A5 and MYC, mTOR and ATF4 (*p* < 0.001) and the positive relationship between MYC, HIF2A and SLC7A5 was only observed in luminal B tumours (*p* = 0.01 and *p* < 0.001, respectively).

**Table 5:** Correlation of *SLC7A5* expression with the expression of other related genes in the Molecular Taxonomy of Breast Cancer International Consortium (METABRIC) data

	SLC7A5 mRNA expression									
	All cases (*n* = 1980)		Luminal A (*n* = 368)		Luminal B (*n* = 367)		HER2+ (*n* = 110)		Triple negative (*n* = 150)	
	Correlation Coefficient (*p* value)			Adjusted *p* value						
Regulatory and other associated genes										
MYC	0.133 (2.4 × 10^−9^)	**<0.0001**	0.012 (0.752)	4.145	0.155 (0.001)	**0.019**	0.066 (0.310)	4.650	0.103 (0.062)	0.434
mTOR	0.085 (0.0001)	**0.001**	−0.005 (0.904)	1.824	0.088 (0.052)	0.728	−0.023 (0.723)	5.784	0.067 (0.226)	1.130
VEGFA	0.352 (6.4 × 10^−59^)	**<0.0001**	0.166 (0.000008)	**0.0002**	0.260 (5.3 × 10^−9^)	**<0.0001**	0.269 (0.00002)	**0.0005**	0.244 (0.000008)	**0.0002**
HIF2A	−0.050 (0.028)	0.168	−0.023 (0.536)	4.896	0.215 (0.000002)	**<0.0001**	0.112 (0.083)	1.328	−0.282 (1.8 × 10^−7^)	**<0.0001**
ATF4	0.159 (1.0 × 10^−12^)	**<0.0001**	− 0.029 (0.433)	5.100	0.057 (0.208)	2.080	0.143 (0.026)	0.468	0.108 (0.050)	0.450
Glutamine-proline regulatory axis										
GLS	0.048 (0.032)	0.192	0.008 (0.829)	3.428	0.055 (0.222)	1.998	−0.006 (0.927)	4.635	−0.115 (0.038)	0.456
ALDH4A1	−0.053 (0.019)	0.133	0.018 (0.638)	4.512	0.063 (0.163)	1.793	−0.028 (0.663)	5.967	−0.134 (0.015)	0.225
PRODH	0.004 (0.858)	1.716	−0.034 (0.369)	4.763	−0.032 (0.483)	2.415	0.037 (0.573)	5.73	0.030 (0.582)	1.746
PYCR1	0.32 (1.5 × 10^−50^)	**<0.0001**	0.143 (0.0001)	**0.001**	0.253 (1.3 × 10^−8^)	**<0.0001**	0.210 (0.001)	**0.024**	0.303 (1.9 × 10^−8^)	**<0.0001**
ALDH18A1	0.222 (1.6 × 10^−23^)	**<0.0001**	0.151 (0.00004)	**0.0008**	0.144 (0.001)	**0.018**	0.168 (0.009)	0.180	0.356 (2.9 × 10^−11^)	**<0.0001**
GLUL	−0.18 (3.3 × 10^−16^)	**<0.0001**	0.134 (0.0003)	**0.005**	0.008 (0.863)	1.726	−0.001 (0.991)	1.982	−0.122 (0.028)	0.392
GLUD1	−0.38 (4.3 × 10^−69^)	**<0.0001**	− 0.161 (0.00001)	**0.0002**	− 0.237 (1.1 × 10^−7^)	**<0.0001**	−0.148 (0.022)	0.418	−0.112 (0.042)	0.420
Glutamine/glutamate transporters										
SLC1A5	0.29 (4.5 × 10^−41^)	**<0.0001**	0.170 (0.000005)	**0.0001**	0.150 (0.001)	**0.017**	0.208 (0.001)	**0.023**	0.25 (0.000002)	**<0.0001**
SLC3A2	0.17 (1.1 × 10^−14^)	**<0.0001**	0.067 (0.072)	3.780	0.193 (0.00001)	**0.0002**	0.184 (0.004)	0.084	0.158 (0.004)	0.064
SLC6A19	0.004 (0.869)	1.738	0.041 (0.273)	4.428	−0.008 (0.859)	2.577	0.047 (0.473)	5.676	−0.103 (0.061)	0.488
SLC7A6	0.362 (2.7 × 10^−62^)	**<0.0001**	0.254 (5.3 × 10^−12^)	**<0.0001**	0.33 (2.0 × 10^− 14^)	**<0.0001**	0.284 (0.000008)	**0.0002**	0.071 (0.201)	1.206
SLC7A7	0.19 (4.6 × 10^−19^)	**<0.0001**	0.007 (0.857)	2.712	0.085 (0.061)	0.793	0.041 (0.530)	5.830	−0.22 (0.00005)	**0.0001**
SLC7A8	−0.42 (1.1 × 10^−88^)	**<0.0001**	−0.115 (0.002)	**0.034**	−0.103 (0.022)	0.352	−0.203 (0.002)	**0.044**	−0.40 (3.9 × 10^− 14^)	**<0.0001**
SLC7A9	− 0.068 (0.002)	0.01	0.025 (0.510)	4.82	0.044 (0.333)	2.331	−0.123 (0.056)	0.952	0.283 (1.8 × 10^−7^)	**<0.0001**
SLC38A1	−0.10 (0.000003)	**<0.0001**	−0.041 (0.270)	3.549	0.039 (0.391)	2.346	0.053 (0.413)	5.369	0.113 (0.041)	0.451
SLC38A2	−0.055 (0.015)	0.120	−0.074 (0.048)	1.05	−0.103 (0.022)	0.330	0.007 (0.917)	5.502	−0.119 (0.032)	0.416
SLC38A3	0.18 (8.3 × 10^−17^)	**<0.0001**	0.140 (0.0001)	**0.002**	0.046 (0.311)	2.488	0.003 (0.958)	2.874	0.196 (0.0003)	**0.005**
SLC38A5	0.011 (0.627)	2.574	−0.069 (0.066)	1.08	−0.077 (0.090)	1.080	−0.017 (0.793)	5.551	−0.017 (0.757)	1.514
SLC38A7	0.306 (3.8 × 10^−44^)	**<0.0001**	0.270 (2.0 × 10^−13^)	**<0.0001**	0.32 (1.2 × 10^−13^)	**<0.0001**	0.064 (0.324)	4.536	0.177 (0.001)	**0.017**
SLC38A8	0.023 (0.312)	1.560	−0.019 (0.612)	4.466	0.011 (0.801)	3.204	−0.006 (0.930)	3.720	−0.039 (0.482)	1.928

*p* value in bold in these tables means statistically significant associations

High SLC7A5 mRNA expression was specifically associated with the enzymes involved with conversion of glutamine (Gln) to proline, where PYCR1 and ALDH18A1 showed a positive relationship with SLC7A5 in almost all subtypes (*p* < 0.02).

The majority of glutamine transporters were significantly associated with SLC7A5 expression primarily in triple negative tumours and to a lesser extent luminal and HER2+ subtypes. SLC7A5 was significantly correlated with SLC1A5 in all subtypes (*p* < 0.02).

*TP53* mutations were also highly prevalent in breast tumours where there was high SLC7A5 mRNA expression (*p* < 0.001, Tables 3 and 4). Moreover, high SLC7A5 protein was positively associated with high p53 protein (*p* < 0.001).

SLC7A5 protein expression was significantly expressed in breast tumours with high Ki67, and the upstream effector MYC (*p* < 0.001, Table 6). SLC1A5, GLS, PYCR1 and PIK3CA were significantly expressed in breast tumours with high expression of SLC7A5 (*p* < 0.001), while the low expression of SLC7A5 was associated with high levels of p-mTORC1 (*p* < 0.001) (Table 6).

**Table 6:** Correlation between SLC7A5 protein expression and other biomarkers in the discovery set

SLC7A5 protein			
Biomarker	Correlation coefficient	*p* value	Adjusted *p* value
c-MYC	0.164	8.2 × 10^−7^	**<0.0001**
Ki67	0.311	1.1 × 10^−21^	**<0.0001**
P-mTORC1	−0.150	0.00001	**<0.0001**
PIK3CA	0.190	3.4 × 10^−7^	**<0.0001**
SLC1A5	0.331	1.1 × 10^−25^	**<0.0001**
GLUD1	0.053	0.09	0.180
GLS	0.371	2.2 × 10^−30^	**<0.0001**
PYCR1	0.283	1.07 × 10^−16^	**<0.0001**

*p* value in bold in these tables means statistically significant associations

### SLC7A5 expression and patient outcome

**Fig. 4:**
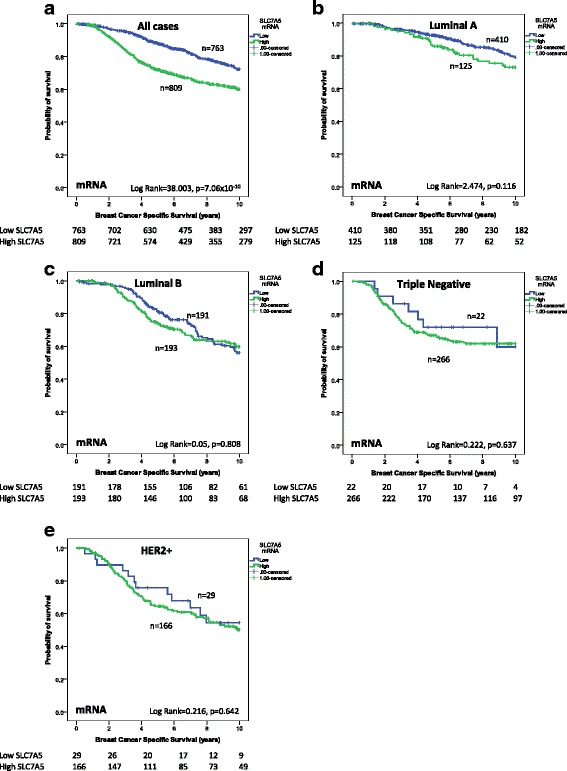
SLC7A5 mRNA and breast cancer patient outcome. **a** SLC7A5 vs breast-cancer-specific survival (BCSS) in all cases. **b** SLC7A5 vs BCSS in luminal A tumours. **c** SLC7A5 vs BCSS in luminal B tumours. **d** SLC7A5 vs BCSS in triple negative tumours. **e** SLC7A5 vs BCSS in human epidermal growth factor receptor 2 (HER2+) tumours

**Fig. 5:**
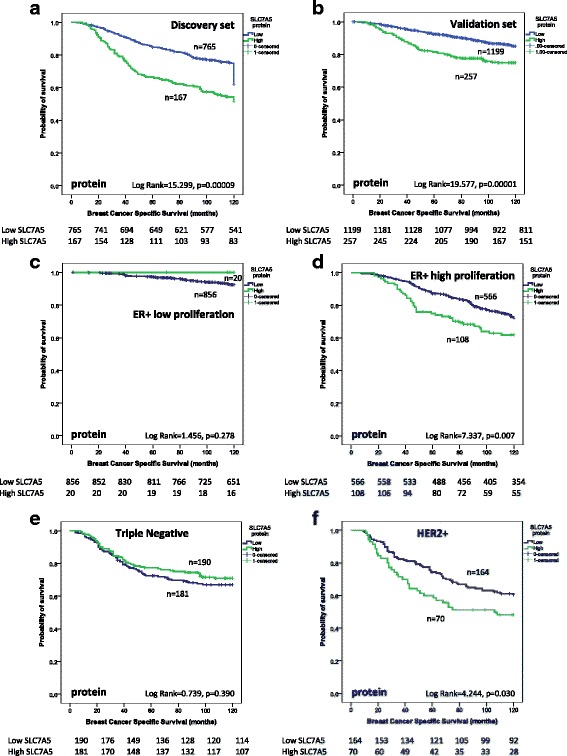
SLC7A5 and breast cancer patient outcome. **a** SLC7A5 vs breast-cancer-specific survival (BCSS) in all discovery set cases. **b** SLC7A5 vs BCSS in all validation set cases. **c** SLC7A5 vs BCSS in oestrogen receptor (ER) + low proliferation tumours in the combined discovery and validation cases. **d** SLC7A5 vs BCSS in ER + high proliferation tumours in the combined discovery and validation cases. **e** SLC7A5 vs BCSS of triple negative tumours in the combined discovery and validation cases. **f** SLC7A5 vs BCSS human epidermal growth factor receptor 2 (HER2)+ tumours in the combined discovery and validation cases

Both high SLC7A5 mRNA (*p* < 0.001) (Fig. 4a) and protein (*p* < 0.001) expression were associated with poor BCSS in the discovery and validation sets (Fig. 5a, b). This association was anticipated as the cutoff was based on the prediction of BCSS.

While SLC7A5 mRNA expression was not predictive of BCSS in any specific molecular class (Fig. 4b-e), high expression of SLC7A5 protein was only predictive of shorter BCSS in ER+ high proliferation (*p* = 0.007, Fig. 5d) and HER2+ tumours (*p* = 0.03, Fig. 5f). There was no association between SLC7A5 protein and outcome in ER+ low proliferation (Fig. 5c) or in TNBC (Fig. 5e). In multivariate Cox regression analysis, SLC7A5 mRNA was a predictor of shorter BCSS independent of tumour size, grade or lymph node stage (*p* = 0.006, Additional file 5: Table S3) but not in any specific subtype. However, SLC7A5 protein was significant only in the ER+ high-proliferation tumours (*p* = 0.02, Table 7) and not in any other subtypes (data not shown).

**Table 7:** SLC7A5 protein expression and patient outcome in the combined discovery and validation sets in all cases and in ER-positive high proliferation tumours

SLC7A5 protein						
	All cases			ER+ high proliferation		
Parameter	Hazard ratio (95% CI)	*p* value	Adjusted *p* value	Hazard ratio (95% CI)	*p* value	Adjusted *p* value
SLC7A5	1.001 (1.000–1.003)	0.063	0.126	1.004 (1.001–1.006)	0.006	**0.024**
Lymph node stage	2.060 (1.813–2.341)	1.7 × 10^−28^	**<0.0001**	1.756 (1.427–2.161)	1.04 × 10^−7^	**<0.0001**
Size	1.365 (1.111–1.678)	0.003	**0.009**	1.169 (0.838–1.632)	0.358	0.716
Grade	2.454 (2.023–2.977)	1.8 × 10^−20^	**<0.0001**	1.756 (1.154–2.672)	0.009	**0.027**

*p* value in bold in these tables means statistically significant associations

Likewise, high SLC7A5 protein expression was associated with shorter distant metastases-free survival (DMFS) (*p* < 0.001; Additional file 6: Figure S2A, B) within the ER+ high-proliferation class (*p* = 0.03, Additional file 6: Figure S2D) but not in other subtypes (Additional file 6: Figure S2C, E, F). This association was identified in the discovery set and validated in the validation set**.** The relationship between high SLC7A5 mRNA expression and poor patient outcome in ER+ disease, but not ER- disease, was confirmed using Breast Cancer Gene-Expression Miner (Additional file 7: Figure S3A, B, C).

## Discussion

Breast cancer is a heterogeneous disease with various subtypes [42] differing in terms of morphology, molecular and biological profiles, response to therapy and clinical behaviour. In addition, different subtypes exhibit disparity in their metabolic pathways and their nutritional needs. The most common form of BC (~ 55–80%) is the ER+/luminal tumour [43, 44], and tumours that belong to this class are also variable in terms of recurrence, mortality rates and disease prognosis [43]. Therefore, understanding the biology of BC and exploring the metabolic pathways could help to identify potential novel therapeutic targets.

Cancer cells must alter their metabolism in order to satisfy the demands of necessary energy and cellular building blocks. It is widely known that amino acid transport systems play a principal role in sustaining the proliferation of cancer cells by supplying the required amino acids for protein synthesis and by activation of nutrient signalling through the mTORC1 complex. This study has revealed for the first time that SLC7A5 is a key amino acid transporter in the more aggressive and highly proliferative ER+ tumours.

*SLC7A5* is located in 16q24.2. This study showed that *SLC7A5* deletion, but not amplification, was significantly correlated with all the annotated genes located in the same chromosomal region, indicating that the deletion was not locus-specific. Interestingly, E-cadherin (*CDH1*), which was located in 16q22.1, was also implicated. It is widely known that most lobular tumours harbour loss of heterozygosity (LOH) at chromosome 16 and are missing the wild type *CDH1* allele [45]. In this study, approximately 40% of METABRIC cases with *SLC7A5* loss were associated with invasive lobular histology. In addition, SLC7A5 protein expression in lobular carcinoma has a relatively lower mean rank value compared to the other histological subtypes, confirming that deletions involve large segments of q16, which can reflect the BC phenotype.

SLC7A5 is widely expressed in many human cancers and various cancer cell lines [46]. The current study included two large discovery and validation cohorts to confirm the significant association between the high SLC7A5 protein expression and the poor prognostic clinico-pathological parameters, including larger tumour size, higher grade and poor NPI. Furthermore, high SLC7A5 expression was significantly associated with higher expression of the proliferative marker (Ki67). This supports the results of previous studies which demonstrated that these two biomarkers are significantly correlated in tongue cancer [47], neuroendocrine carcinoma of the lung [48], thymic carcinoma [49] and breast cancer [50], indicating that SLC7A5 is critical for proliferation in cancer cells.

With respect to the breast cancer ER+ subtypes, SLC7A5 expression was lower in ER+ tumours that have low proliferation (luminal A subtype) compared with the highly proliferative ER+ (luminal B) malignancies, and it was primarily associated with poor patient outcome and shorter DMFS in the latter class only. This is most likely due to their heavier energy and nutrient requirements for cell survival, proliferation and metastasis. This was anticipated, as it has been shown that over expression of SLC7A5 is actively involved in the proliferation of vascular smooth muscle cells [51] and it is co-expressed with vascular endothelial growth factor (VEGF) in the primary and metastatic sites of many cancers [37], which may be implicated for the metastatic process. In this study the most significant positive correlation between mRNA expression of SLC7A5 and VEGFA was identified in the luminal B subtype. In this regard, Bartlett et al. included SLC7A5 as a part of the five-gene Mammostrat® immunohistochemistry panel, where the higher expression is used to predict recurrence-free survival (RFS), DMFS and overall survival (OS) in ER+ breast cancer during endocrine therapy [52]. However, they did not consider the different molecular subtypes of BC.

SLC7A5 mRNA and protein was also highly expressed in TNBC and HER2+ BC, in concordance with Furuya et al. [50]. However, in these subtypes the significant association between SLC7A5 protein expression and patient outcome was only observed in the HER2+ tumours. Among all BC subtypes, SLC7A5 protein expression was an independent predictor of short BCSS in ER+ high-proliferation tumours only. In this regard, the larger sample of ER+ high-proliferation cases might reflect the stronger significance compared with the smaller sample of HER2+ and TNBC tumours. We therefore suggest that further confirmation in larger cohorts of HER2+ and TN tumours is warranted.

Previous studies have shown regulation of SLC7A5 by other proteins including the tumour oncogene Myc, which induces SLC7A5 [4, 5]. In the current study, the relationship between SLC7A5 and other regulatory proteins in both mRNA and protein expression was investigated. A positive relationship was observed between SLC7A5 and Myc in both protein and mRNA levels, and this correlation remained significant only in luminal B subtype, when different subtypes were investigated. ATF4-dependent transcripts also encode for SLC7A5 upon amino acid deprivation [39] and in this study there was positive correlation between ATF4 and SLC7A5 gene expression, in line with expectations. A previous study showed that activation of the HIF2α pathway increases mTORC1 activity by upregulating expression of the amino acid carrier SLC7A5 [38] and the current study confirmed the positive correlation between HIF2α and SLC7A5, which was only observed in luminal B tumours. SLC7A5 functions by importing essential amino acids to cancer cells and research has detailed the role of amino acids, particularly leucine, in activating mTORC1, which in turn controls protein translation and cell proliferation, and prevents apoptosis in malignant cells [2, 3]. This study showed positive correlation between SLC7A5 and mTOR at the mRNA level. However, there were conflicting results in the analysis of protein levels of SLC7A5, whereby high SLC7A5 expression was associated with lower expression of the mTORC1 phosphorylated at ser (2448), which was included in this study. This was unsurprising as Cheng et al. confirmed that phosphorylation of mTORC1 at ser (2448), which is stimulated by growth factors, was mutually exclusive with mTORC1 phosphorylated at thr (2446), which is regulated by amino acids [53]. These observations may explain why SLC7A5 over expression is primarily associated with poor outcome only in the high proliferation ER+ tumours.

This study further investigated the association of SLC7A5 expression with other glutamine transporters, in which some variability in the expression of amino acid transporters across molecular subtypes was observed. For example, the TN subtype was the main class associated with the transporters required for glutamine influx, perhaps because it depends on delivery of glutamine instead of synthesis. In contrast, positive correlation between SLC7A5 and the glutamine synthase enzyme GLUL was observed in luminal A tumours, suggesting that this subtype might rely on glutamine neosynthesis rather than uptake. SLC1A5 functionally couples with SLC7A5 to allow the cellular influx and efflux of glutamine, as SLC1A5 mediates uptake of glutamine, while SLC7A5 uses intracellular glutamine concentrations to adjust the essential amino acid cytoplasmic pool for metabolic demands and signalling to mTORC1 [15]. Here we observed that SLC7A5 and SLC1A5 are positively correlated in all the BC subtypes.

Previous studies have raised awareness and revealed the importance of the proline-glutamine (Pro-Gln) regulatory axis in BC. SLC7A5 appears to have a pivotal role in this regulatory axis, as its expression was highly associated with the enzymes that mediate glutamate degradation to form the amino acid proline, which has been shown to play a role in assisting tumour growth by different mechanisms [54].

Blocking of SLC7A5 using its inhibitor, 2-aminobicyclo-(2,2,1)-heptane-2-carboxylic acid (BCH), efficiently decreased colony formation of MDA-MB-231 TNBC cells [55]. Even though the consequences of blocking SLC7A5 in the highly proliferative ER+ tumours remain undetermined, this study suggests that SLC7A5 can potentially be used as a therapeutic target for luminal B BC**.**

## Conclusion

This study revealed and confirmed that the glutamine transporter SLC7A5 is associated with poor prognostic characteristics and poor survival outcome. Over expression of SLC7A5 appears to play a role in the proliferation and progression of the aggressive ER+ subtype of breast cancer, thus it could act as a potential therapeutic target. Functional assessment is necessary to reveal the specific role played by this amino acid transporter in the highly proliferative subclass and HER2+ BC.

## Additional files

Additional file 1: **Table S1.** Clinicopathological parameters of the METABRIC and Nottingham discovery and validation series. (DOCX 16 kb)

Additional file 2: **Figure S4.** SLC7A5 mRNA expression, in the TCGA data, and its association with copy number alteration (A), staging system (B), ER status (C), PR status (D) and HER2 (E) status. (PPTX 176 kb)

Additional file 3: **Table S2.** List of genes with copy number loss that was significantly associated with *SLC7A5* deletion in the METABRIC and TCGA data. (DOCX 16 kb)

Additional file 4: **Figure S1.** SLC7A5 gene expression and its association, using Breast Cancer Gene-Expression Miner v4.0, with: tumour grade (A), NPI (B), ER status (C), PR status (D), HER2 status (E), Triple Negative status (F)and PAM50 subtypes (G). (PPTX 117 kb)

Additional file 5: **Table S3.** SLC7A5 mRNA and patient outcome. (DOCX 14 kb)

Additional file 6: **Figure S2.** SLC7A5 vs DMFS in all cases in the discovery set (A), all cases in the validation set (B), ER + low proliferation tumours in the combined discovery and validation set cases (C), ER + high proliferation tumours in the combined discovery and validation set cases (D), triple negative tumours in the combined discovery and validation set cases (E) and HER2+ tumours in the combined discovery and validation set (F). (PPTX 407 kb)

Additional file 7: **Figure S3.** SLC7A5 mRNA and breast cancer patient outcome using Breast Cancer Gene-Expression Miner in unselected cases (K), ER+ disease (L) and ER- disease (M). (PPTX 80 kb)

## Abbreviations

BC: Breast cancer
BCH: 2-Aminobicyclo-(2,2,1)-heptane-2-carboxylic acid
BCSS: Breast cancer specific survival
CDH1: E-cadherin
CISH: Chromogenic in situ hybridisation
CK: Cytokeratin
CNA: Copy number aberration
CNV: Copy number variation
DM: Distant metastasis
DMFS: Distant metastasis-free survival
ECL: Enhanced chemiluminescence
ER: Oestrogen receptor
Gln: Glutamine
HER2: Human epidermal growth factor receptor 2
kDa: KiloDalton
LN: Lymph node
LOH: Loss of heterozygosity
METABRIC: Molecular Taxonomy of Breast Cancer International Consortium
mTORC1: Mammalian target of rapamycin complex1
NPI: Nottingham Prognostic Index
PAM50: Prediction analysis of microarray 50
PBS: Phosphate-buffered saline
PgR: Progesterone receptor
Pro: Proline
SNP: Single nucleotide polymorphism
TBS: Tris-buffered saline
TCGA: The Cancer Genome Atlas
TMA: Tissue microarray
TN: Triple negative
TNBC: Triple negative breast cancer
VEGF: Vascular endothelial growth factor
