# Supplementary material for: The amino acid transporter SLC7A5 confers a poor prognosis in the highly proliferative breast cancer subtypes and is a key therapeutic target in luminal B tumours
Source: Breast Cancer Res. 2018 Mar 22;20:21. doi: 10.1186/s13058-018-0946-6 (PMC5863851; doi:10.1186/s13058-018-0946-6)
Supplement: Supplementary file 1 — Table S1. Clinicopathological parameters of the METABRIC and Nottingham discovery and validation series. (DOCX 16 kb) [file 13058_2018_946_MOESM1_ESM.docx]

**Table S1.** Clinicopathological parameters of the METABRIC and Nottingham discovery and validation series.

|  | **METABRIC series**  **n (%)** | **Nottingham discovery set**  **n (%)** | **Nottingham**  **Validation set**  **n (%)** |
| --- | --- | --- | --- |
| **Age** |  |  |  |
| ≥ 50 years | 1426 (78.6) | 714 (61.3) | 1070 (69.5) |
| ˂ 50 years | 424 (21.4) | 395 (38.7) | 469 (30.5) |
| **Tumour size** |  |  |  |
| ≥ 2cm | 1337 (68.2) | 577 (52.0) | 599 (38.9) |
| ˂2cm | 623 (31.8) | 532 (48.0) | 939 (61.1) |
| **Grade** |  |  |  |
| 1 | 169 (9.0) | 190 (17.2) | 231 (15.0) |
| 2 | 770 (40.7) | 366 (33.1) | 622 (40.4) |
| 3 | 952 (50.3) | 550 (49.7) | 685 (44.5) |
| **Tumour type** |  |  |  |
| Ductal (including mixed) | 1545 (83.6) | 922 (83.1) | 1335 (86.9) |
| Lobular | 148 (8.0) | 101 (9.1) | 120 (7.8) |
| Medullary-like | 32 (1.7) | 26 (2.3) | 13 (0.8) |
| Miscellaneous | 12 (0.6) | 7 (0.6) | 9 (0.6) |
| Special type | 113 (6.1) | 53 (4.9) | 60 (3.9) |
| **Vascular Invasion** |  |  |  |
| Definite | Not available | 382 (34.6) | 451 (29.3) |
| Negative/Probable |  | 722 (65.4) | 1086 (70.7) |
| **Lymph Node Stage** | | |  |
| 1 | 1035 (52.5) | 674 (70.0) | 955 (62.2) |
| 2 | 623 (31.5) | 341 (30.8) | 428 (27.9) |
| 3 | 315 (16.0) | 91 (8.2) | 153 (10.0) |
| **Follow-up Status** |  |  |  |
| Alive | 1070 (55.7) | 569 (51.3) | 1110 (72.2) |
| Died from Breast Cancer | 505 (26.3) | 365 (33.0) | 282 (18.3) |
| Died from other causes | 345 (18.0) | 175 (15.7) | 146 (9.5) |
| **Estrogen Receptor** |  |  |  |
| Negative | 472 (23.8) | 270 (24.6) | 300 (19.5) |
| Positive | 1508 (76.2) | 827 (75.4) | 1240 (80.5) |
| **Progesterone Receptor** |  |  |  |
| Negative | 938 (47.4) | 435 (40.8) | 612 (41.8) |
| Positive | 1042 (52.6) | 630 (59.2) | 853 (58.2) |
| **HER2 status** |  |  |  |
| Negative | 1734 (87.5) | 921 (86.6) | 1376 (89.9) |
| Positive | 246 (12.5) | 143(13.4) | 155 (10.1) |
