# Supplementary material for: The amino acid transporter SLC7A5 confers a poor prognosis in the highly proliferative breast cancer subtypes and is a key therapeutic target in luminal B tumours
Source: Breast Cancer Res. 2018 Mar 22;20:21. doi: 10.1186/s13058-018-0946-6 (PMC5863851; doi:10.1186/s13058-018-0946-6)
Supplement: Supplementary file 2 — Figure S4. SLC7A5 mRNA expression, in the TCGA data, and its association with copy number alteration (A), staging system (B), ER status (C), PR status (D) and HER2 (E) status. (PPTX 176 kb) [file 13058_2018_946_MOESM2_ESM.pptx]

## Slide 1
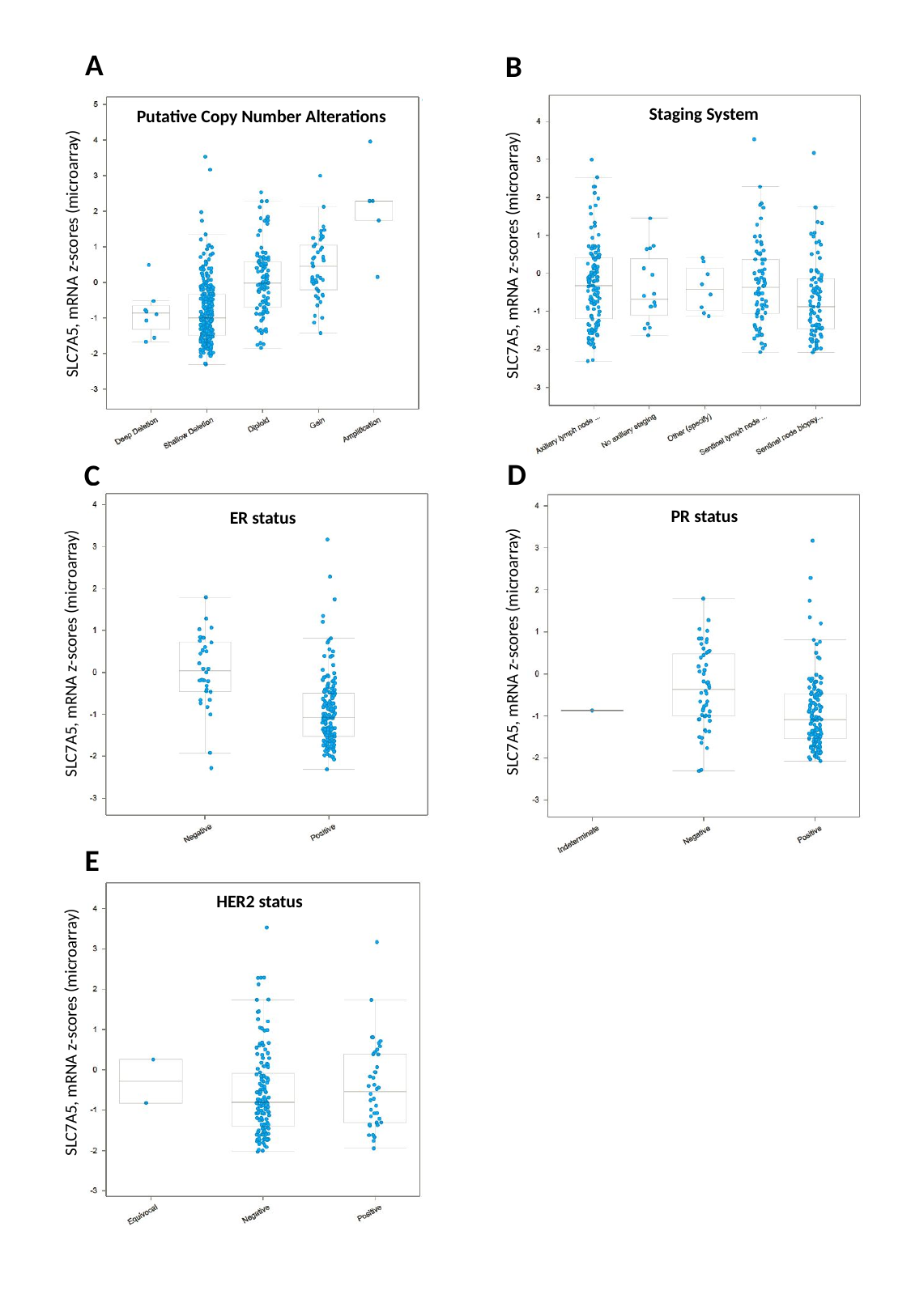

A
B
Staging System
Putative Copy Number Alterations
SLC7A5, mRNA z-scores (microarray)
SLC7A5, mRNA z-scores (microarray)
D
C
PR status
ER status
SLC7A5, mRNA z-scores (microarray)
SLC7A5, mRNA z-scores (microarray)
E
HER2 status
SLC7A5, mRNA z-scores (microarray)
