# Supplementary material for: The amino acid transporter SLC7A5 confers a poor prognosis in the highly proliferative breast cancer subtypes and is a key therapeutic target in luminal B tumours
Source: Breast Cancer Res. 2018 Mar 22;20:21. doi: 10.1186/s13058-018-0946-6 (PMC5863851; doi:10.1186/s13058-018-0946-6)
Supplement: Supplementary file 3 — Table S2. List of genes with copy number loss that was significantly associated with SLC7A5 deletion in the METABRIC and TCGA data. (DOCX 16 kb) [file 13058_2018_946_MOESM3_ESM.docx]

**Table S2**. List of genes whose copy number loss was significant associatedwith *SLC7A5* deletion in the METABRIC and TCGA data.

| **Gene symbol** | **Description** | **Chromosomal location** | **TCGA**  **P-value** | **METABRIC**  **P-value** |
| --- | --- | --- | --- | --- |
| *SIAH1* | siah E3 ubiquitin protein ligase 1 | 16q12.1 | 1.5x10^-3^ | 1.3x10^-3^ |
| *ABCC11* | ATP binding cassette subfamily C member 11 | 16q12.1 | 1.5x10^-3^ | 1.3x10^-3^ |
| *CYLD* | CYLD lysine 63 deubiquitinase | 16q12.1 | 1.5x10^-3^ | 1.3x10^-3^ |
| *MMP2* | matrix metallopeptidase 2 | 16q12.2 | 1.2x10^-4^ | 1.8x10^-4^ |
| *RBL2* | RB transcriptional corepressor like 2 | 16q12.2 | 6.1x10^-3^ | 4.3x10^-4^ |
| *AMFR* | autocrine motility factor receptor | 16q13 | 3.1x10^-5^ | 1.8x10^-4^ |
| *MMP15* | matrix metallopeptidase 15 | 16q21 | 1.2x10^-4^ | 1.8x10^-4^ |
| *USB1* | U6 snRNA biogenesis phosphodiesterase 1 | 16q21 | 1.2x10^-4^ | 1.8x10^-4^ |
| *CX3CL1* | C-X3-C motif chemokine ligand 1 | 16q21 | 1.2x10^-4^ | 1.8x10^-4^ |
| *NOL3* | nucleolar protein 3 | 16q22.1 | 8.8x10^-7^ | 3.3x10^-3^ |
| *NQO1* | NAD(P)H quinone dehydrogenase 1 | 16q22.1 | 2.3x10^-8^ | 2.2x10^-4^ |
| *TERF2* | telomeric repeat binding factor 2 | 16q22.1 | 8.8x10^-7^ | 8.02x10^-3^ |
| *CBFB* | core-binding factor beta subunit | 16q22.1 | 8.8x10^-7^ | 6.1x10^-3^ |
| *CDH1* | cadherin 1 | 16q22.1 | 1.2x10^-5^ | 3.4x10^-4^ |
| *CDH3* | cadherin 3 | 16q22.1 | 4.3x10^-6^ | 1.4x10^-4^ |
| *CTCF* | CCCTC-binding factor | 16q22.1 | 8.8x10^-7^ | 1.3x10^-5^ |
| *E2F4* | E2F transcription factor 4 | 16q22.1 | 8.8x10^-7^ | 9.4x10^-6^ |
| *PHLPP2* | PH domain and leucine rich repeat protein phosphatase 2 | 16q22.2 | 1.3x10^-7^ | 2.2x10^-6^ |
| *ZFHX3* | zinc finger homeobox 3 | 16q22.2-16q22.3 | 1.3x10^-7^ | 1.1x10^-6^ |
| *BCAR1* | BCAR1, Cas family scaffolding protein | 16q23.1 | 3.8x10^-9^ | 1.8x10^-5^ |
| *WWOX* | WW domain containing oxidoreductase | 16q23.1-16q23.2 | 3.7x10^-8^ | 6.3x10^-6^ |
| *ATMIN* | ATM interactor | 16q23.2 | 1.2x10^-11^ | 3.1x10^-5^ |
| *MAF* | MAF bZIP transcription factor | 16q23.2 | 9.5x10^-11^ | 4.5x10^-7^ |
| *OSGIN1* | oxidative stress induced growth inhibitor 1 | 16q23.3 | 3.8x10^-11^ | 1.1x10^-7^ |
| *CDH13* | cadherin 13 | 16q23.3 | 2.1x10^-12^ | 1.1x10^-6^ |
| *FOXF1* | FOXF1 adjacent non-coding developmental regulatory RNA | 16q24.1 | 2.4x10^-13^ | 1.1x10^-7^ |
| *WFDC1* | WAP four-disulfide core domain 1 | 16q24.1 | 2.1x10^-12^ | 1.1x10^-6^ |
| *FBXO31* | F-box protein 31 | 16q24.2 | 2.4x10^-13^ | 1.1x10^-7^ |
| *FANCA* | Fanconi anemia complementation group A | 16q24.3 | 2.1x10^-12^ | 5.4x10^-4^ |
| *CBFA2T3* | CBFA2/RUNX1 translocation partner 3 | 16q24.3 | 2.1x10^-12^ | 7.8x10^-5^ |
| *CDT1* | chromatin licensing and DNA replication factor 1 | 16q24.3 | 2.1x10^-12^ | 7.8x10^-5^ |
