# Supplementary figures and images for: The amino acid transporter SLC7A5 confers a poor prognosis in the highly proliferative breast cancer subtypes and is a key therapeutic target in luminal B tumours
Source: Breast Cancer Res. 2018 Mar 22;20:21. doi: 10.1186/s13058-018-0946-6 (PMC5863851; doi:10.1186/s13058-018-0946-6)

## Slide 1
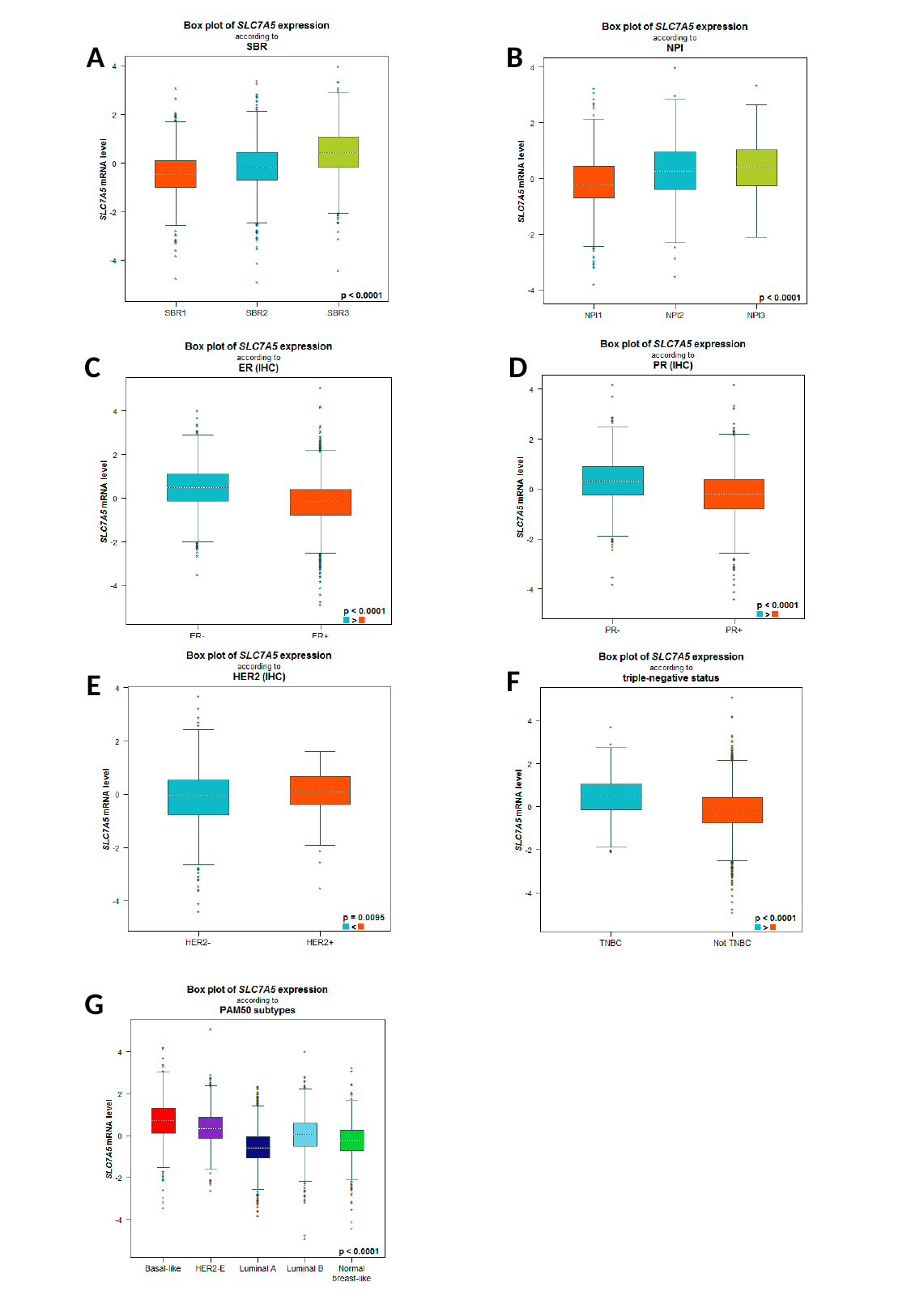

A
B
C
D
F
E
G

Supplement: Supplementary file 4 — Figure S1. SLC7A5 gene expression and its association, using Breast Cancer Gene-Expression Miner v4.0, with: tumour grade (A), NPI (B), ER status (C), PR status (D), HER2 status (E), Triple Negative status (F)and PAM50 subtypes (G). (PPTX 117 kb) [file 13058_2018_946_MOESM4_ESM.pptx]

## Slide 1
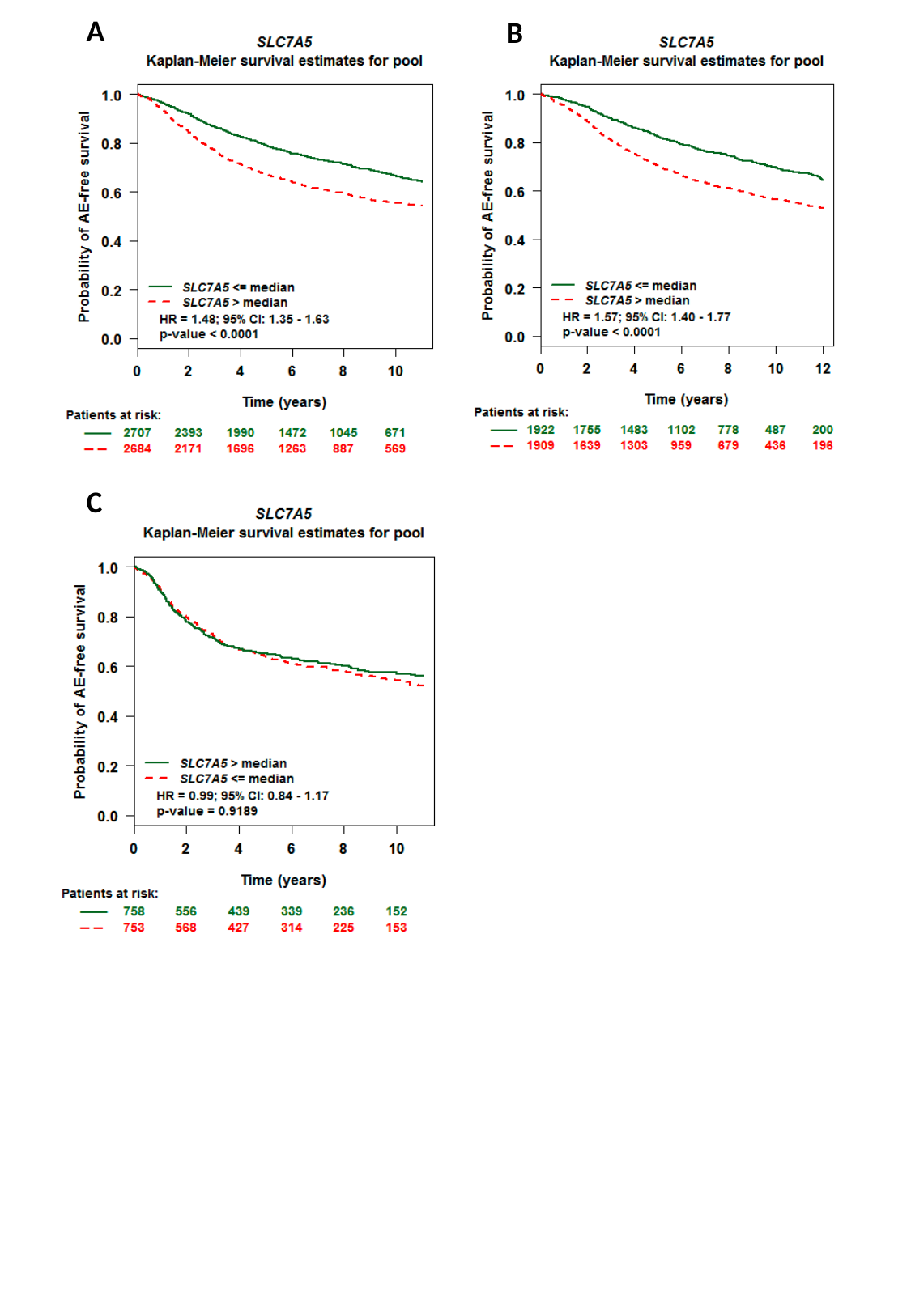

A
B
C

Supplement: Supplementary file 7 — Figure S3. SLC7A5 mRNA and breast cancer patient outcome using Breast Cancer Gene-Expression Miner in unselected cases (K), ER+ disease (L) and ER- disease (M). (PPTX 80 kb) [file 13058_2018_946_MOESM7_ESM.pptx]
