# Supplementary material for: The amino acid transporter SLC7A5 confers a poor prognosis in the highly proliferative breast cancer subtypes and is a key therapeutic target in luminal B tumours
Source: Breast Cancer Res. 2018 Mar 22;20:21. doi: 10.1186/s13058-018-0946-6 (PMC5863851; doi:10.1186/s13058-018-0946-6)
Supplement: Supplementary file 5 — Table S3. SLC7A5 mRNA and patient outcome. (DOCX 14 kb) [file 13058_2018_946_MOESM5_ESM.docx]

**Table S3**. SLC7A5 mRNA and patient outcome.

| **SLC7A5 mRNA** | | | |
| --- | --- | --- | --- |
| **Parameter** | **Hazard ratio**  **(95% CI)** | **p-value** | **Adjusted p-value** |
| **SLC7A5** | 1.121 (1.039-1.209) | 0.003 | **0.006** |
| **LN stage** | 1.877 (1.656-2.129) | 8.9x10^-23^ | **<0.0001** |
| **Size** | 1.550 (1.219-1.971) | 0.0003 | **0.001** |
| **Grade** | 1.331 (1.108-1.599) | 0.002 | **0.006** |
