# Supplementary material for: The amino acid transporter SLC7A5 confers a poor prognosis in the highly proliferative breast cancer subtypes and is a key therapeutic target in luminal B tumours
Source: Breast Cancer Res. 2018 Mar 22;20:21. doi: 10.1186/s13058-018-0946-6 (PMC5863851; doi:10.1186/s13058-018-0946-6)
Supplement: Supplementary file 6 — Figure S2. SLC7A5 vs DMFS in all cases in the discovery set (A), all cases in the validation set (B), ER + low proliferation tumours in the combined discovery and validation set cases (C), ER + high proliferation tumours in the combined discovery and validation set cases (D), triple negative tumours in the combined discovery and validation set cases (E) and HER2+ tumours in the combined discovery and validation set (F). (PPTX 407 kb) [file 13058_2018_946_MOESM6_ESM.pptx]

## Slide 1
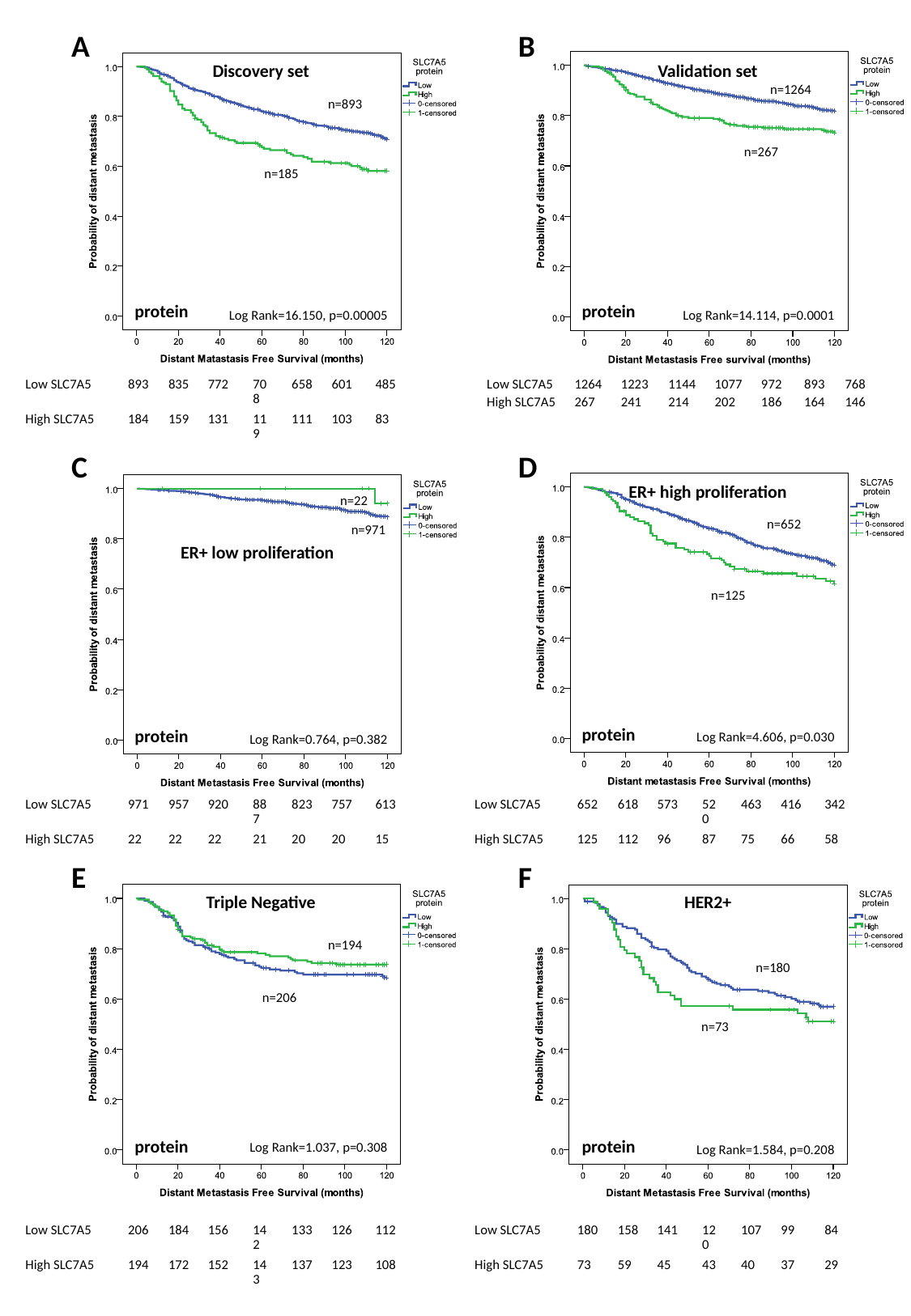

A
Discovery set
n=893
n=185
protein
Log Rank=16.150, p=0.00005
B
Validation set
n=1264
n=267
protein
Log Rank=14.114, p=0.0001
| Low SLC7A5 | 893 | 835 | 772 | 708 | 658 | 601 | 485 |
| --- | --- | --- | --- | --- | --- | --- | --- |
| High SLC7A5 | 184 | 159 | 131 | 119 | 111 | 103 | 83 |
| Low SLC7A5 | 1264 | 1223 | 1144 | 1077 | 972 | 893 | 768 |
| --- | --- | --- | --- | --- | --- | --- | --- |
| High SLC7A5 | 267 | 241 | 214 | 202 | 186 | 164 | 146 |
C
n=22
n=971
ER+ low proliferation
protein
Log Rank=0.764, p=0.382
D
ER+ high proliferation
n=652
n=125
protein
Log Rank=4.606, p=0.030
| Low SLC7A5 | 971 | 957 | 920 | 887 | 823 | 757 | 613 |
| --- | --- | --- | --- | --- | --- | --- | --- |
| High SLC7A5 | 22 | 22 | 22 | 21 | 20 | 20 | 15 |
| Low SLC7A5 | 652 | 618 | 573 | 520 | 463 | 416 | 342 |
| --- | --- | --- | --- | --- | --- | --- | --- |
| High SLC7A5 | 125 | 112 | 96 | 87 | 75 | 66 | 58 |
E
Triple Negative
n=194
n=206
protein
Log Rank=1.037, p=0.308
F
HER2+
n=180
n=73
protein
Log Rank=1.584, p=0.208
| Low SLC7A5 | 206 | 184 | 156 | 142 | 133 | 126 | 112 |
| --- | --- | --- | --- | --- | --- | --- | --- |
| High SLC7A5 | 194 | 172 | 152 | 143 | 137 | 123 | 108 |
| Low SLC7A5 | 180 | 158 | 141 | 120 | 107 | 99 | 84 |
| --- | --- | --- | --- | --- | --- | --- | --- |
| High SLC7A5 | 73 | 59 | 45 | 43 | 40 | 37 | 29 |
